# Photocatalytic Activity of Thin Layers Obtained via Electrodeposition and Annealing of Nanostructured WFeZn and WFeCu Alloys

**DOI:** 10.1021/acsomega.4c11131

**Published:** 2025-05-15

**Authors:** Tomasz Ratajczyk, Krzysztof Miecznikowski, Pawel Majewski, Rafal Maciag, Mikolaj Donten

**Affiliations:** 49605 University of Warsaw, Faculty of Chemistry, Pasteura 1, Warsaw 02-093, Poland

## Abstract

Improvement of the efficiency of the water-splitting process is one of the crucial issues to be dealt with in the coming years. In this study, a new method for the preparation of photocatalysts is presented. Two novel light-activated oxygen evolution catalysts were developed, consisting of oxidized forms of tungsten, iron, and zinc or copper. In the two-step synthesis, thin layers of nanostructured tungsten–iron–third metal alloys are electrodeposited from an aqueous bath initially, and then they are annealed in an oxidizing atmosphere. The electroplating technique was used in the designed process to combine high precision in deposition and control of composition with relatively low economic and environmental costs. In addition, the easier formation of highly active tungstate forms in the catalysts may be favored by the structure of the alloy precursors. Conditions for obtaining the layers were optimized based on recorded photocurrents. The analysis of electrode surfaces was performed using spectroscopic and microscopic techniques. The oxygen released during the photooxidation of water with ternary metal oxide semiconductors was monitored using an O_2_ membrane sensor (Clark electrode), and the conversion efficiency was found to be approximately 30%.

## Graphical abstract

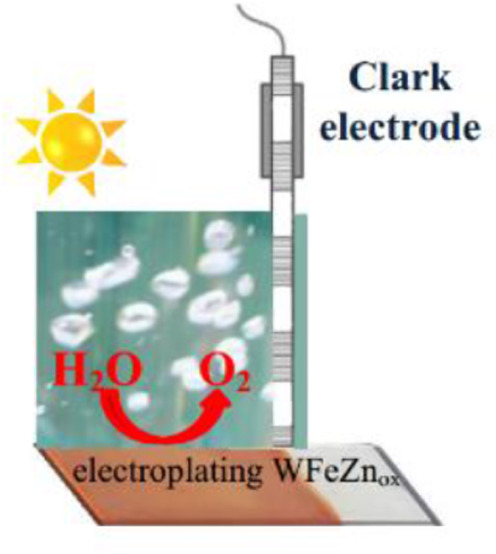

## Introduction

Solar irradiation is undoubtedly the largest source of renewable energy among all other nonconventional energy sources. The sunlight energy delivered to the Earth during 1 h could provide the annual energy demand of the entire human population. However, in order to utilize solar energy and become completely independent of fossil fuels, technologies and materials must be developed to absorb, convert, and store solar energy in a way that is independent of the Sun’s diurnal cycle.

Semiconductor photocatalytic technology, as a green and efficient method, can be applied for absorbing visible light energy directly at ambient temperature. Solar energy can be used for splitting water into its constituent elements,^1^ reducing carbon dioxide^2,3^ and degrading organic pollutants and dye waste without inducing secondary pollution.^4,5^ Among all semiconductor metal oxides, five metal oxides are important and extensively studied photocatalytic materials: TiO_2_,^6,7^ WO_3_,^8,9^ Fe_2_O_3_,^10,11^ Cu_2_O^12^, and ZnO.^13^ The semiconductor may be n-type, p-type, or a coupling of n-type and p-type forming a heterojunction. Despite the many benefits of utilizing single-component semiconductor materials as photocatalysts, a major drawback arises from the fact that they do not exhibit the necessary properties for optimal photocatalytic performance. The crucial properties include strong visible light absorption, conduction and valence band edges straddling the reduction or oxidation potentials (e.g., water), rapid interfacial charge transfer, and high recombination efficiency of photoinduced charge carriers.^14^ One of the promising approaches proposed in the literature to restrain these drawbacks is to produce binary, ternary or multinary metal compounds,^15–17^ such as Ag_3_PO_4_/CuBi_2_O_4_,^18^ MoS_2_/graphene,^19^ Ag/AgCl/TiO_2_,^20^ WO_3_/Fe_2_O_3_,^21,22^ and BiOBr/BiOI/Fe_3_O_4_ ^23^ with enhanced photocatalytic properties. All of these show a higher photocatalytic efficiency in comparison to single-component semiconductors. Forming semiconductor/semiconductor heterojunction structures by linking multimetal oxides is a well-known approach to enhance photoelectrochemical properties. The heterojunction structures between various semiconductors with matched band energy are capable of shifting the absorption wavelength as well as improving photoelectrochemical performance due to the separation of photogenerated electron–hole pairs between semiconductors with diverse energy levels. The modification of morphologies in heterojunction structures usually improves photocatalytic performance in comparison to single-semiconductor metal oxides. It is worth noting that the connection of different metal oxide semiconductors with diverse energy levels forms a system with prompt charge separation and a reduced recombination rate of generated electron–hole pairs, leading to improved photocatalytic activity for water splitting or for photodegradation of organic pollutants in aqueous environments.

Through the last decades, many different synthesis procedures for semiconducting materials have been described in the literature, such as coprecipitation, plasma, sonochemical precipitation, thermal oxidation, chemical etching, pulsed spin coating,^24^ spray-pyrolysis deposition (SPD),^25^ hydrothermal methods,^26^ sol–gel routes,^8^ ion-assisted sputtering, liquid phase deposition (LPD), ion implantation, and chemical vapor deposition (CVD).^27^ Utilizing these procedures leads to the generation of different structures, e.g., nanospheres, nanorods, or nanosheets. Among these methods, the sol–gel, coprecipitation, and sonochemical precipitation techniques exhibit the required merits, such as ease of preparation and the lack of necessity for sophisticated equipment for the synthesis of nanostructured photocatalytic materials. Galvanic methods based on electrodeposition processes have also been used for the formation of semiconductor layers.^28,29^ However, so far, the strategies of electrodeposition often involve obtaining materials directly from relatively scarce or toxic precursors,^30^ not taking into account the simpler possibility of electrodeposition of metals or their alloys as intermediate products.

Electrodeposition of metals from aqueous solutions, i.e. plating baths, is a cost-efficient strategy, as it usually does not involve expensive reagents or advanced instruments. Thus, as long as it does not exploit toxic components, it can be considered green. Galvanic methods of material synthesis allow for obtaining certain homogeneous alloys consisting of immiscible components that cannot be achieved by other methods. Another important advantage of the electroplating strategy is the relatively good control of layer parameters, especially thickness. Although electrodeposition of tungsten–iron alloys from aqueous solutions has been researched for almost a hundred years,^31,32^ this process has not yet been deliberately utilized for obtaining mixed oxide photocatalysts, e.g., for OER. However, metallic layers of tungsten alloys have been considered very efficient HER electrocatalysts.^33,34^ Conversion of electrodeposited tungsten alloys to oxides has been investigated, but primarily as a study of the alloy properties.^35^ While research has mostly focused on the electrodeposition of binary tungsten alloys, there are also studies on ternary systems, mainly with copper as the third component.^36^ Nonetheless, the particular tungsten–iron–copper alloy has not been investigated yet. There is a recent report on the electrodeposition of a tungsten–iron–zinc alloy, which emphasized the anticorrosive properties of the alloy coating.^37^ Among the multiple parameters influencing the result of the electrodeposition process are plating bath temperature, cathodic current density during electroplating,^38^ and the concentration of bath components. The latter also includes low-concentration additives that strongly influence the composition and morphology of the deposit, such as butynediol, which acts as an inhibitor and surface brightener.^39^

The aim of the present study is that the ternary metal oxide semiconductor layer, prepared by electrocodeposition of ternary alloys followed by annealing under an oxygen atmosphere, yields a material that exhibits photocatalytic activity. Cognizant of the potential importance of the selection of metal oxide semiconductors, the hypothesis was tested on two alloys: tungsten–iron–zinc (labeled WFeZn) as well as tungsten–iron–copper (labeled WFeCu). To differentiate between the alloys and their oxidation products, the oxidized materials are sublabeled “ox”. Among important issues, the coexistence of single metal oxides and ferric and zinc tungstates^35^ after annealing cannot be excluded, especially given that the homogeneous structure of the electrodeposited alloy should favor the synthesis of tungstates at relatively low temperatures. To the best of our knowledge, so far, there has been no attempt to utilize a conventional metal electroplating method for the synthesis of such photocatalytic materials.

## Experimental Section

### Reagents

The chemicals were analytical-grade reagents; they were used as received. Exact reagent names and their concentrations are provided in the following chapter. Aqueous solutions were prepared using double-distilled and subsequently deionized (Millipore Milli-Q) water. Conductive glass slides (F-doped SnO_2_ – FTO, *R* = 7 Ω/square, Sigma-Aldrich), as well as Cu and Ag sheets, were used as substrates. FTO was utilized as the substrate because of its higher stability at excessively negative values of applied potential during electrodeposition compared to ITO. The FTO substrates, prior to each electrodeposition, were successively washed with ethanol, then cleaned electrochemically by passing +25 mA/cm^2^ anodic current for 1.5 min in Na_2_CO_3_ cleaning bath, and then thoroughly washed again with water.

### Plating Bath Preparation

The ternary layers of WFeZn and WFeCu alloys were electrodeposited from citrate-tungstate-based plating baths. Typical plating baths contained the following main components: trisodium citrate dihydrate, 81.6 g·dm^–3^; disodium tungstate dihydrate 79 g·dm^–3^; boric acid 10.5 g·dm^–3^; 85% phosphoric acid, 6.1 cm^3^·dm^–3^. The baths also included additions of 1,4-butynediol (in various concentrations) as a brightener and 70 ppm of nonoxynol-10 as the surface agent. Lastly, various amounts of metal ion sources were added to the solutions, i.e., ferric ammonium citrate and copper sulfate or zinc sulfate. The use of ferric ammonium citrate instead of widely used ferrous salts was chosen mainly due to the stability of iron­(III) in comparison to iron­(II), which oxidizes in aqueous solutions, and also due to iron­(III) being already complexed by citrates in the utilized compound.

### Electrodeposition and Thermal Conversion

All coatings were prepared in an electrolytic cell with separate cathode and anode compartments containing different electrolytes. The cathode compartment was filled with the plating bath solution, while a 0.25 M sodium sulfate solution was used as the anode electrolyte to avoid decomposition of the bath components.

Deposition parameters such as bath temperature, plating current density, duration of deposition, and metal ion concentrations were optimized in the first stage of experiments. The samples were deposited at a constant current of −18, −35, −70, or −105 (±2) mA/cm^2^ with potentiostat/galvanostat EG&G PAR 173A. The plating system was maintained at a constant temperature by using a thermostatic water bath. As starting parameters, thin layers were deposited at 65 (±1)°C for 30 min on metals and 90 (±1) s on FTO, as precursors for photocatalytic materials. During the optimization process, the bath temperature was set to 25, 45, 65, or 85 (±1)°C, and the deposition time ranged from 15 s to 15 min. In plating baths for both materials, iron­(III), zinc­(II), and copper­(II) ions were present in the following concentrations: 18, 27, and 36 mM Fe^3+^; 1, 2, and 4 mM Zn^2+^ for WFeZn; and 0.5, 1, and 2 mM Cu^2+^ for WFeCu. The samples for further characterization were deposited under the optimal conditions, as detailed in the results section.

Samples of alloys were also formed as galvanic coatings on the copper substrate for WFeZn and on the silver substrate for WFeCu for composition analysis. To obtain photoactive materials, the samples of the electroplated alloys were placed into a quartz tube furnace and annealed in pure oxygen at 600 °C for 60 min, to convert the metallic elements into their oxidized forms. Although a higher annealing temperature would seem to promise even better efficiency of the catalyst, heating the samples above 650 °C results in severe damage to the glass substrate.

### Analytical Methods

SEM-EDX analysis of the deposited layers and the ensuing ternary metal oxide layers was conducted for the observation of surface morphology and the determination of sample composition. FE-SEM Zeiss Merlin was used for sample imaging, and sample composition was analyzed with the Bruker Quantax 400 EDX detector. For elemental analysis, thicker alloy coatings deposited on metallic surfaces were chosen, as the layers on FTO were too thin to provide reliable quantitative results.

UV–vis spectra were recorded using a Jasco V-650 spectrophotometer equipped with a 60 mm integrating sphere (Jasco, Easton, MD, USA). Spectra were recorded in reflectance mode and are presented as normalized absorbance. Utilizing the resulting data, the band gaps of the ternary metal oxide semiconductor films were estimated using Tauc plots, where the occurrence of indirect allowed transitions was assumed.

Photoelectrochemical measurements were performed in a “cappuccino cell” in the three-electrode configuration, in which the counter electrode was made from a carbon rod, the working electrode utilized the deposited metal oxide semiconductor film on FTO, and the reference electrode was K_2_SO_4_-saturated Hg/HgSO_4_ (MSE). Herein, all potentials are expressed vs the reversible hydrogen electrode (RHE). Photoanodes were irradiated from the side of the photoactive material layer/solution interface, and the exposed electrode surface area was 0.28 cm^2^. Photoelectrochemical experiments were carried out under simulated AM 1.5 G solar irradiation (Newport 81094 Model) at a scan rate of 10 mV·s^–1^ in 0.5 mol·dm^–3^ H_2_SO_4_ using a CH Instruments Model 760E Electrochemical Workstation. During measurements, light intensity was adjusted to 100 mW·cm^–2^ using the calibrated reference cell (Portable Radiometer, International Light Technologies Model 1400 with SEL623) and thermopile detector (with NIST Traceable Calibration). The irradiation steps were interrupted by using a manual chopper. The incident photon-to-current conversion efficiencies (IPCE) recorded vs excitation wavelength were obtained by irradiating the samples with a 500 W xenon lamp and utilizing a Multispec Model 257 monochromator (Oriel) with a 4 nm bandwidth. To assess the role of active species (such as holes (h^+^) and electron (e^–^)) created in photocatalytic reactions, miscellaneous scavengers were employed. Here, isopropyl alcohol, ethylenediaminetetraacetic acid disodium (EDTA-Na), and potassium bromate (KBrO_3_) were applied as OH, hole, and electron scavengers, respectively.

XRD analyses of the samples, both metallic and thermally oxidized, were performed by using a powder X-ray diffractometer (D8 Discover, Bruker Inc.) equipped with a collimated Cu Kα radiation (0.154 nm) source. The data were collected in the 10°–100° 2θ range, with a 0.01° step size, in a locked-coupled mode using a 1D linear detector (Vantec). X-ray diffraction (PXRD) analysis and data fitting were conducted using Topas software (Bruker Inc.).

Raman spectra were obtained using a DXR Raman spectrometer (Thermo Scientific) equipped with a 50x/NA 0.5 objective and a laser set to 532 nm. To evaluate sample uniformity, the spectra were collected from various sites on the surface.

## Results and Discussion

### Electrodeposition Optimization

Both the alloys themselves and their application for photoelectrocatalysis are still a novelty. Therefore, at the beginning, it was crucial to examine the influence of the conditions of their preparation on the photocatalytic properties. The optimization of the process for obtaining WFeZn and WFeCu layers included several parameters. These parameters were as follows: the quantitative composition of the baths, regarding the concentrations of iron­(III), zinc­(II), and copper­(II) salts, as well as butynediol; deposition current density; bath temperature; and the duration of electrodeposition.

For each parameter, the series of annealed samples of WFeZn_ox_ and WFeCu_ox_ were then examined with linear sweep voltammetry (LSV). Parameters that provided the highest photocurrent density on the prepared sample were considered optimal and conserved in the following sample series. The diagnostic parameters were the recorded photocurrent and the onset potential of the photocatalytic process. The suitable recorded photocurrent–potential curves for a series of samples are presented in Figures S1, S3, and S4.

Given the number of parameters that require optimization, the deposition of ternary alloys instead of simpler binary tungsten–iron could be questioned. However, adding the third metal to the alloy resolved two important problematic issues associated with WFe. The oxidized binary WFe_ox_ layer was not observed to work properly as an OER photocatalyst; instead, on the LSV plot for WFe, only a slight and erratic current increase could be observed, without a significant current increase during the illumination intervals. In contrast, the electrocatalytic activity of the WFeZn_ox_ and WFeCu_ox_ samples increased strongly during illumination and rose with WE potential, as shown in Figure 1. The relevant photocurrent was observed only during the illuminated intervals. The photocurrent increased somewhat concavely across the entire potential range.

**Figure 1:**
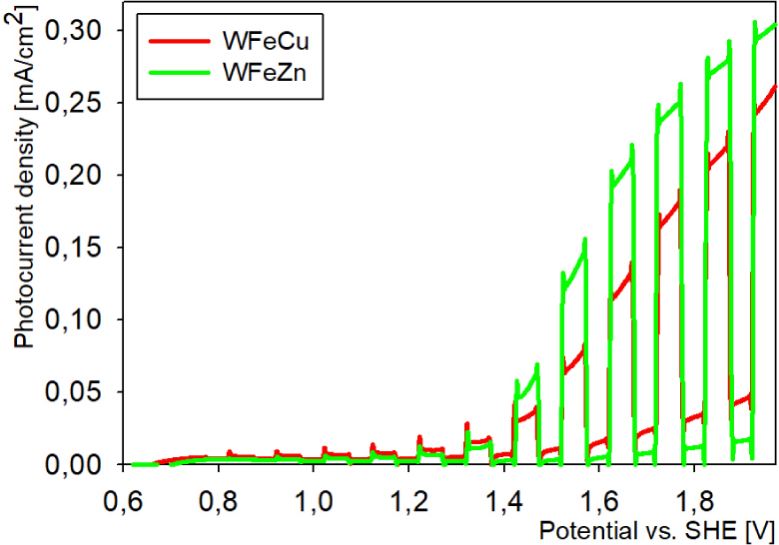
Photocurrent comparison for WFeZn_ox_ and WFeCu_ox_.

Thus, introducing copper or zinc into the WFe alloys enhances the photocatalytic performance of these materials after annealing. Among these two systems, WFeZn_ox_ yields higher photocurrents—this result will be described more precisely later on. Moreover, it was observed that a small percentage of either zinc or copper in the WFe alloy coating significantly boosted its corrosion resistance in air. As a result, after annealing, WFeZn_ox_ and WFeCu_ox_ tend to be more uniform than binary WFe layers, which had already been partially corroded before the annealing.

The choice of the optimal concentrations of 27 mM Fe^3+^, 2 mM Zn^2+^, and 1 mM Cu^2+^, respectively, was based on the photocurrent–voltage curves for the obtained materials, attached in Figure S1.

It should also be noted that the presence of zinc ions in the bath solution remarkably inhibits tungsten electrodeposition, the more the Zn^2+^ concentration is. This results in both a reduction in the tungsten content in the deposit and a decrease in the overall rate of alloy deposition. A similar effect, though somewhat weaker, is observed for copper ions, which also reduce the tungsten content in the deposit. It is worth noting that for the WFeZn layers, the best catalyst had a tungsten content of 18 at. %, the highest reported so far. Conversely, for WFeCu, the best catalyst contained the least tungsten, but still as high as 23 at. %. The relevant plots are provided in Figure S2.

In both cases, the best alloy for the catalyst contained approximately 5 at. % of the third metal (Zn or Cu). Generally, the higher the metal ion concentration in the bath, the higher the metal (Fe, Zn, Cu) content in the respective alloys. Tungsten content in the alloy did not correspond clearly to the photocatalytic properties of further oxidized layers, although a maximum of 18% tungsten content in WFeZn and a minimum of 23% tungsten content in WFeCu undeniably provided the highest photocurrent on LSV.

A peculiar observation was made regarding the WFeZn alloy composition. Namely, at a constant Zn^2+^ ion concentration in the bath, the atomic percent of zinc in the deposited alloy seems to be inversely proportional to the concentration of Fe^3+^ ions in the bath. Figure 2 shows this characteristic for three series of baths with a fixed zinc concentration. For each of the series, the product of ferric ion concentration and zinc atomic percent is constant, as represented in the plot (expressed in arbitrary units).

**Figure 2:**
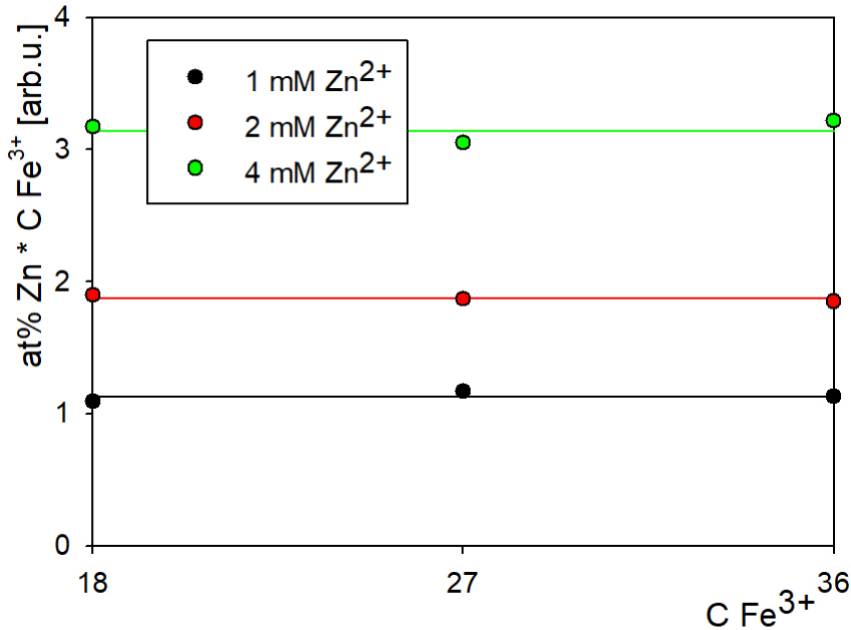
Inverse proportion of iron concentration in bath and zinc content in alloy.

**Figure 3:**
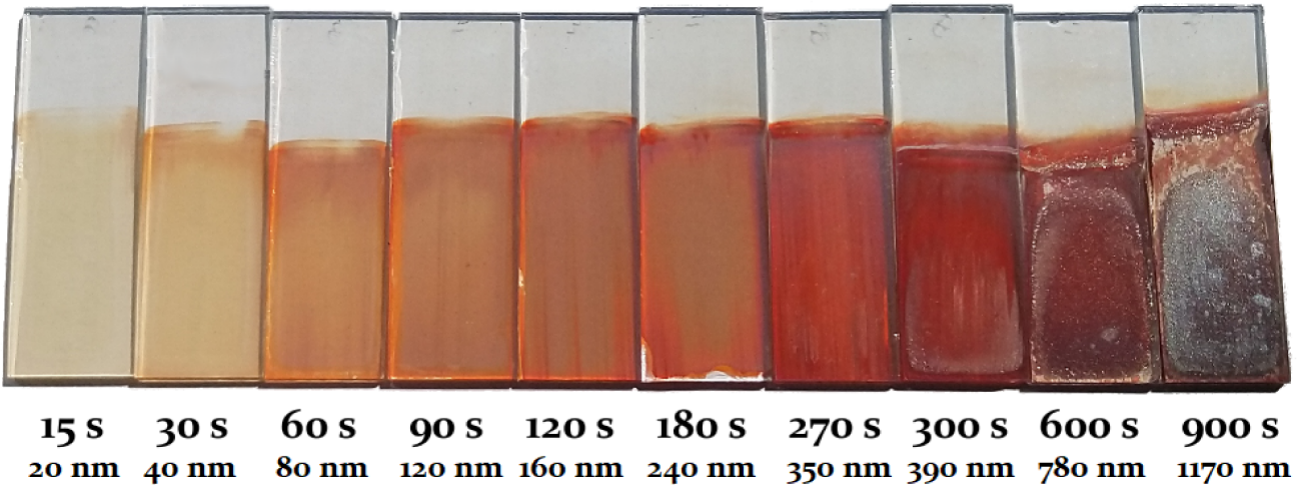
Photographs of WFeZn_ox_ layers by increasing thickness; deposition time and estimated layer thickness are noted below each sample. Photograph taken by the authors.

After choosing the optimal composition of baths, the conditions for the alloys’ electrodeposition were studied, specifically cathodic current density and bath temperature. For each bath, four different current densities (−18, −35, −70, and −105 mA/cm^2^)) were applied to a constant temperature of 65 °C and four different temperatures (25, 45, 65, and 85 °C) were tested for a current density of −35 mA/cm^2^. LSV results for WFeZn_ox_ layers obtained in these conditions show that a current density of −35 mA/cm^2^ and a temperature of 45 °C are optimal. Layers deposited with −70 mA/cm^2^ current density produced roughly half the photocurrent of those deposited at −35 mA/cm^2^, and the remaining layers worked even worse. Among tested bath temperatures, the recorded photocurrents of the layer deposited at 65 and 45 °C were comparable, both higher than those obtained at the lowest (25 °C) and the highest (85 °C) temperatures. Thus, since lowering the bath temperature from 65 to 45 °C does not affect the recorded photocurrent significantly, it allows one to reduce the energy consumed to heat the system roughly by half. In this regard, 45 °C was selected as the optimal temperature to produce the WFeZn_ox_ layers.

In the case of WFeCu_ox_, the final optimal conditions for WFeCu deposition varied significantly more than the initial ones. As already mentioned above, lower tungsten content in this particular alloy tended to give better photocatalytic results when the bath composition was changed. The same effect was observed when changing current density and temperatures in the same manner as for WFeZn. For a constant current density of – 35 mA/cm^2^, the optimal temperature was 25 °C, and deposition of the alloy in higher temperatures yielded materials that exhibited lower photocurrent. Respecting current densities of deposition, for 65 °C, the best current density was as low as −18 mA/cm^2^. Both the lowest current density and lowest bath temperature resulted in low tungsten content, only 10 at. % for 25 °C. Hence, combining both these optimal conditions gave a photocatalytic layer even better than all the previous WFeCu samples. Unexpectedly, the WFeCu alloy deposited at 25 °C, −18 mA/cm^2^ contained as much as 17 at. % tungsten. This observation negates the direct correlation between tungsten content in the alloy and the photocurrent density of the oxidized layer, favoring its structure or morphology instead. Comparisons of the LSV curves of materials derived from WFeZn and WFeCu layers obtained under various temperature and current conditions are provided in Figures S3 and S4.

Some attention was also paid to the influence of butynediol in a bath on the photocatalytic properties of the layer. For both baths with optimal metal ion content, three concentrations of the brightener—0, 50, and 150 mg·dm^–3^—were examined. In general, increased butynediol content slows down the entire electrodeposition process and levels the surface of the coating, i.e., gives the bath brightening properties. It is noteworthy that in the case of WFeZn, upon the introduction of 50 ppm butynediol, the photocurrent recorded on WFeZn_ox_ was slightly increased. However, increasing the brightener concentration up to 150 ppm reduced the photocatalytic activity of the WFeZn_ox_ layer by half. On the other hand, in the case of the WFeCu alloy, the increase in butynediol concentration up to 150 ppm caused an increase in the recorded photocurrent. The influence of the butynediol component in the bath on the morphology of the alloys deposited on the metallic surface was verified using SEM. The images of the WFeCu alloy coatings on the silver substrate are presented in Figure 4. Figure 4 shows the surface of the alloy deposited in the absence (Figure 3a) and presence (Figure 3b) of the brightener (50 ppm butynediol). The sample deposited in the absence of the brightener had many discontinuous defects, as presented in Figure 4a. The images display that the presence of the brightener forms a smoother coating surface but yields a fine-grained (1 μm order) dark powder loosely bound to the surface. EDS analysis of the powder, collected directly after the deposition, showed no remarkable difference in composition between the powder and the main coating. Despite the apparently smoother coating surface, the introduction of butynediol to the bath ultimately increases the overall surface roughness due to the formation of additional structures on the layer surface. As LSV curves of the oxidized layers showed better performance for the layers synthesized in the presence of butynediol, further examination of the alloy layers determined the optimal concentration of the additive to be 50 ppm for WFeZn and 150 ppm for WFeCu. Later on, the diffractograms shown in Figure 6 deny any substantial influence of this bath additive on the crystalline structure of the alloy.

**Figure 4:**
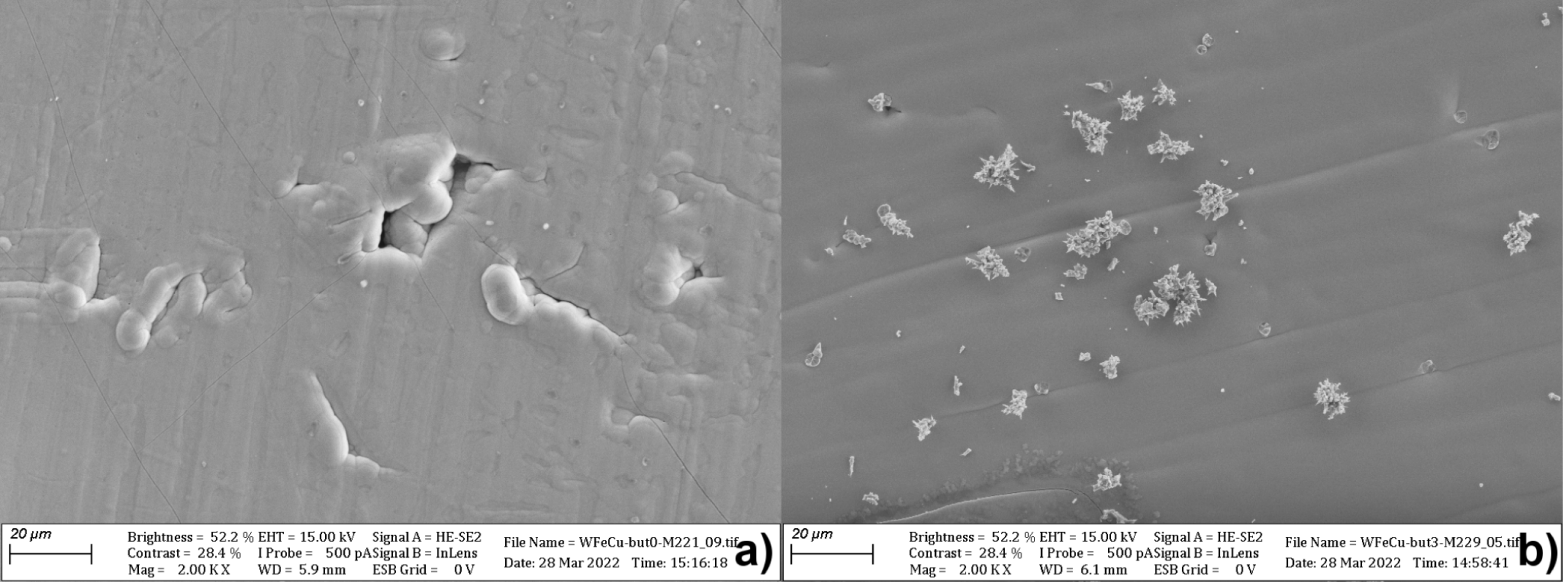
SEM images of WFeCu coating on Ag: (a) without and (b) with 50 mg/L butynediol.

It is also noteworthy that the presence of butynediol in the bath solution leads to a decrease in the electrodeposition efficiency as well as the W content in the obtained alloys. The results imply that the presence of 150 ppm of butynediol in the bath slows the WFeZn alloy deposition rate by three times in comparison to a bath without butynediol (from 7.4 to 2.4 mg·cm^–2^·h^–1^) and decreases the tungsten content in the alloy from 20.3 to 10.4 at. %. The same, but weaker, effect was observed on WFeCu deposits.

It is well known that the photocatalytic properties of the layers strongly depend on their thickness. The determination of the thickness of the films relied on calculating the rate of their growth [μm·cm^–2^·h^–1^] under specific conditions (bath composition, temperature, current density). The layer thickness should then be proportional to the duration of electrodeposition. A series of layers were deposited over various time scales, ranging from 15 to 900 s, covering the entire range of reasonably appropriate thicknesses. Figure 4 shows the influence of the deposition time of the WFeZn layer on the visible thickness of the final product. The thinnest layers, deposited for 15 s, are barely visible. On the other hand, when the electrodeposition time increased to more than 10 min, the annealed layers started to adhere more weakly to the glassy substrate (FTO). The overly thick layers lose their semitransparency, so utilizing them as photocatalytic layers would cease to make sense.

Moreover, a difference in the optimal layer thickness between the two studied alloys was observed. Low temperature and low current density, found to be optimal for the deposition of the WFeCu layer, result in a low rate of layer growth. More precisely, the WFeZn layer grows at approximately 1.3 nm/s, while WFeCu grows at only 0.4 nm/s. WFeZn layers worked properly if their thickness was 80–240 nm, and a 240 nm thick layer, deposited for 180 s, gave the best results for photocatalysis. For WFeCu, the range of proper thickness for the layer was similar, but the optimal thickness was lower, i.e., 130 nm. However, due to the lower growth rate of the WFeCu alloy, deposition of a layer of this thickness requires a longer deposition time, about 300 s. In summary, for the further characterization of photocatalytic materials, the optimal electrodeposition times of 3 and 5 min were selected for WFeZn and WFeCu, respectively.

### Characterization of the Photocatalytic Materials

The morphologies of the photocatalytic materials (WFeZn and WFeCu) were also examined using SEM. Figure 5a displays the SEM image of a representative surface of the metallic WFeZn layer deposited on FTO. The images indicated the presence of nanogranules, where the average size of particles estimated from the SEM images was approximately 100 nm. Furthermore, the size of nanoparticles was observed to be dependent on the time of deposition. The surface morphology of the layers deposited on FTO varies from those deposited on metallic substrates due to the distribution of sites for the metal phase nucleation on rough FTO-coated glass. Heat treatment of both metallic layers does not drastically change the morphology (Figure 5b) and also preserves the size of those objects. On the other hand, their surfaces are slightly rougher due to the formation of metal oxide forms, which considerably favor the catalytic properties of the layers.

**Figure 5:**
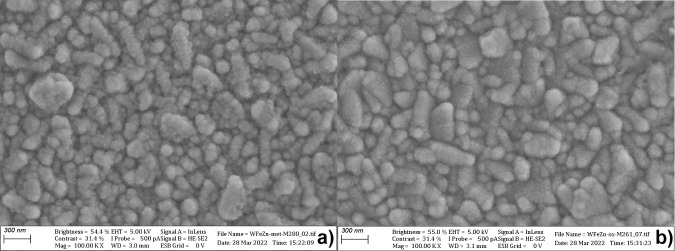
SEM images of WFeZn thin layer surface before (a) and after (b) annealing.

### Physicochemical Characterization of the Photocatalysts

#### X-ray Diffractograms

The structure of the obtained materials was examined by X-ray diffractography. Powder X-ray diffraction (PXRD) analysis of the as-deposited thick (approximately 10 μm) metallic samples deposited on Ag (Figure 6) indicated the presence of poorly crystalline material in both the Zn- and Cu-bearing iron–tungsten alloys, irrespective of the amount of 1,4-butynediol used for electrodeposition. Broad (fwhm ≈ 10° 2θ) reflexes located at ∼44.0° and ∼43.5° (2θ) for the WFeZn and WFeCu alloys, respectively, originate from nanometer-sized crystallites present in these samples. In the WFeCu alloy, they can be attributed to the bcc (110) tungsten (40.4°) and iron (44.6°) reflexes observed in bulk materials, overlapping with the fcc Cu(111) (43.3°). In the WFeZn alloy, the slight shift of the peak’s maximum toward lower angles and an asymmetric shoulder extending <40° would likely originate from hexagonal zinc reflexes (002), (100), and (101) located in the 36–44° 2θ range.

**Figure 6:**
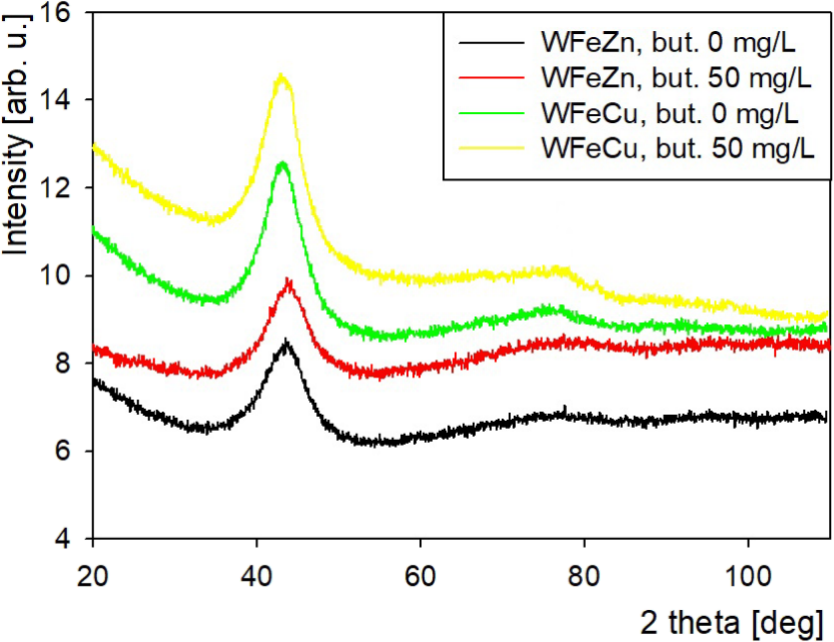
X-ray diffractograms of WFeZn and WFeCu alloys before annealing.

Thermal annealing in pure oxygen at 600 °C for 60 min leads to the oxidation of metals and the formation of distinct crystalline phases. Figure 7 shows a diffractogram of a 240 nm thick WFeZn sample deposited on an FTO-coated glass substrate. In the figure, vertical dashed lines and open symbols mark the reference positions of the crystalline phases identified in the film (red circle – α-Fe_2_O_3_, green triangle – (Zn,Fe)­WO_4_, blue reverse triangle – FTO). The most prominent diffraction peaks (apart from those originating from the FTO), which can be ascribed to the α-Fe_2_O_3_ (hematite) phase, are marked with red circles. The set of less intense reflexes, marked with green triangles, likely originates from isomorphic zinc and iron tungstates ((Fe,Zn)­WO_4_). For the copper-containing layer, a similar graph is shown in Figure S5, along with a more detailed analysis of the diffraction patterns (Figures S6 and S7) and a summary of the crystallographic parameters of the identified material components (Table S1).

**Figure 7:**
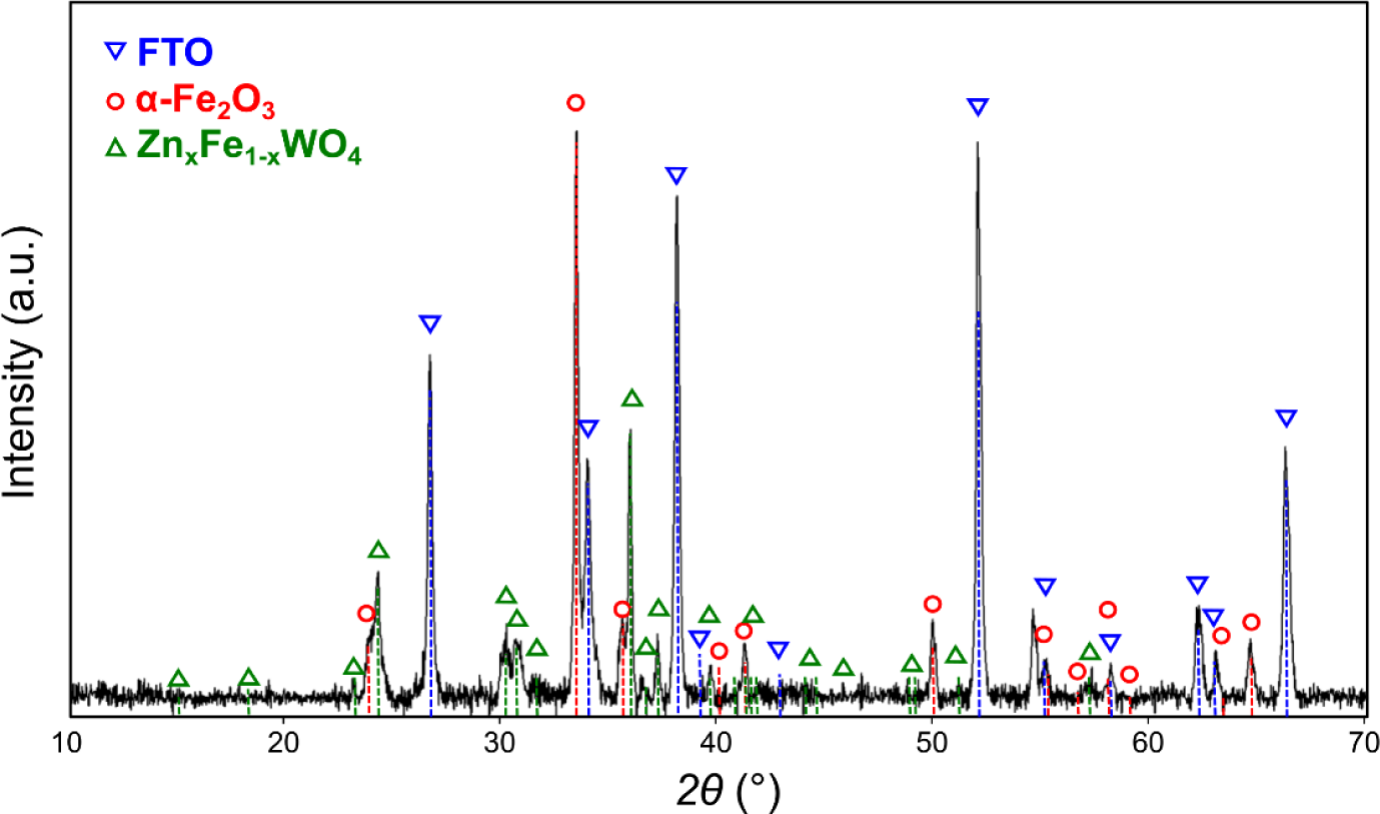
X-ray diffractogram of annealed 240 nm thick WFeZn layer on FTO.

#### Spectrophotometric Measurements

Figure 8 displays the adsorption spectra of all films, namely WFeZn_ox_ and WFeCu_ox_ on FTO electrodes. Both samples exhibited strong absorption in the visible light range, with an onset at around 580 nm. This is related to the electron transition from the valence band to the conduction band. Furthermore, the composite WFeZn_ox_ and WFeCu_ox_ samples demonstrated a noticeably red-shifted absorption onset in comparison to the pristine α-Fe_2_O_3_ film.^40^ Based on these UV–vis data, the optical energy bandgap for both composite samples was determined to be about 2.1 eV for both materials. Tauc plots for the processed spectra are provided in Figure S8. Overall, the optical energy bandgap results demonstrated that the bandgap relative to the pristine metal oxides is very comparable to hematite (2.1 eV), higher than copper oxide (1.2 eV), and lower than tungsten oxide (2.8 eV) and zinc oxide (3.3 eV).^41,42^

**Figure 8:**
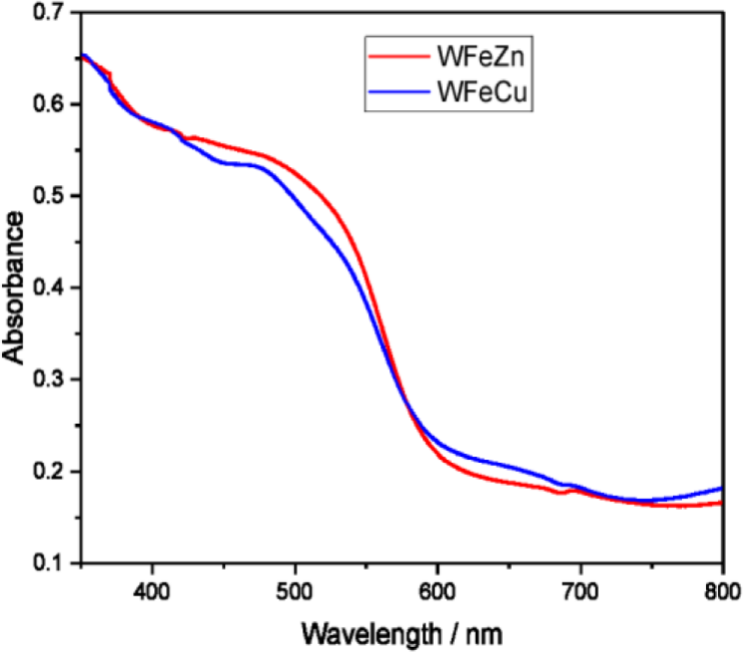
UV–vis absorption spectra of the WFeZn_ox_ and WFeCu_ox_ layers.

#### Raman Analysis

The physicochemistry of both photocatalytic layers was also studied by Raman spectroscopy to confirm the creation of a crystal phase without the presence of residual secondary phases in the pyrolyzed material, such as WO_3_ and other oxides. All spectra (WFeZn_ox_ and WFeCu_ox_) exhibited the following strong bands around 223.18, 242.63, 289.69, 408.48, 494.95, 554 (FTO); 609.15, 660.57, 873.52, 1085.67 (FTO), and 1314.24 cm^–1^. The obtained Raman spectra show seven characteristic phonon lines, namely two modes A1g (224 and 498 cm^–1^) as well as five Eg modes (243, 289, 299, 409, and 608 cm^–1^) indicating the hematite crystalline phase was achieved already in both films, which is visually apparent as well.^43^ Furthermore, the presence of hematite was confirmed by the appearance of one band around 1320 cm^–1^, which should be assigned to a two-magnon scattering. Moreover, the observed strong bands at 554 and 1086 cm^–1^ originated from the FTO substrate. The Raman spectra of both samples (Figure 9ab) do not definitively display vibrational bands coming from other oxides, such as tungsten oxide and zinc oxide for WFeZn_ox_ or tungsten oxide and copper oxide for WFeCu_ox_. This should indicate that tungstate phases could have formed, such as ferric tungstate, zinc tungstate, or copper tungstate.

**Figure 9:**
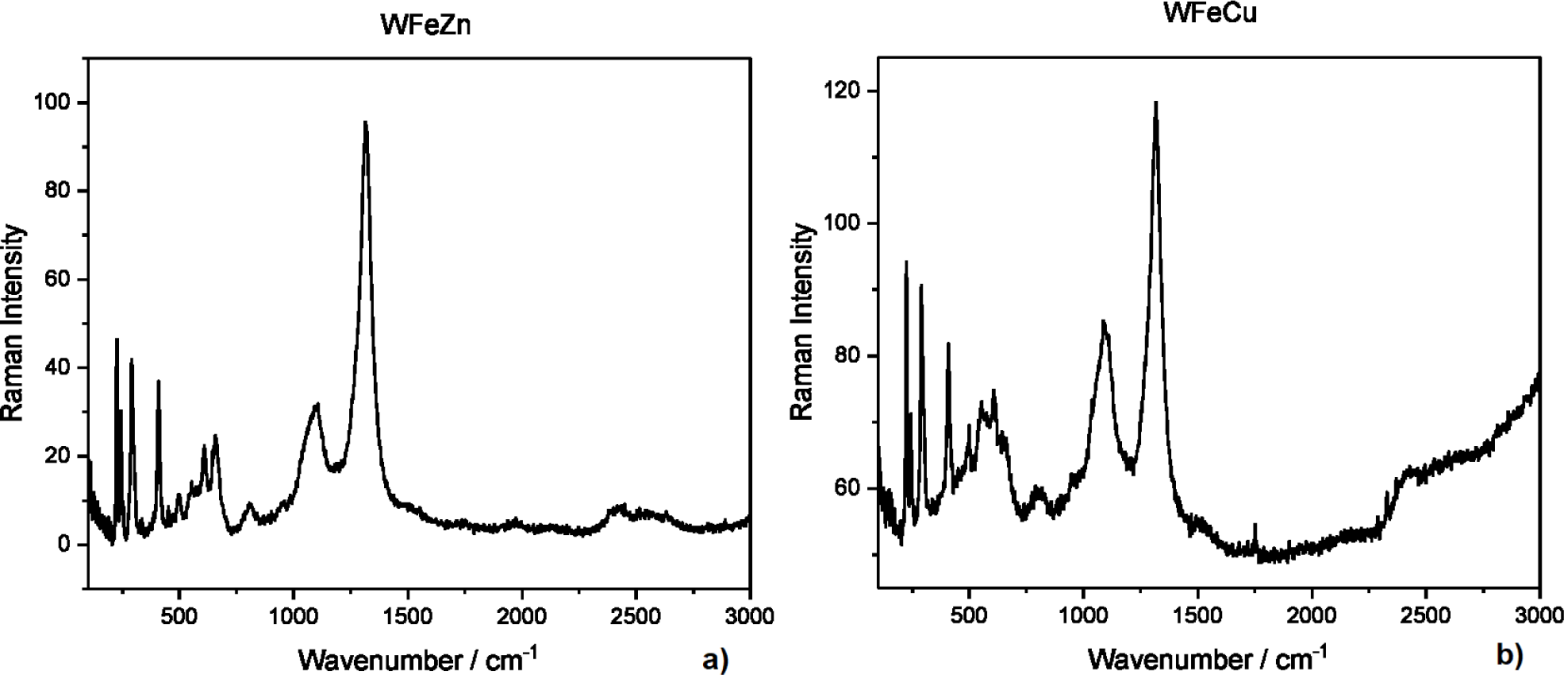
Raman spectra for WFeZn_ox_ (a) and WFeCu_ox_ (b) deposited on FTO.

### Photoelectrochemical Characterization of the Annealed Materials

In order to evaluate the photocatalytic properties of WFeZn_ox_ and WFeCu_ox_, the annealed films were first examined for water photo-oxidation reactions in acidic aqueous solutions. Figure 1, shown earlier in the article, depicts the electrode photocurrent density as a function of applied potential (E) recorded under chopped simulated solar 1.5 AM (100 mW/cm^2^) illumination in acidic media for both alloy films deposited and annealed under optimal conditions. The recorded anodic photocurrents in both systems examined were typical of n-type semiconducting behavior, while the recognized onset potential could be correlated with the flat band potential of the semiconductor electrode, requiring that the interface between the electrolyte and the semiconductor was Schottky-type. The resulting photocurrent densities of both films in acidic media (Figure 1) evidently raised with the increased applied potential. While in the case of WFeZn_ox_, the recorded photocurrent at 1.1 V (i.e., below the thermodynamic potential for oxygen evolution, 1.23 V) approached the level of 0.02 mA/cm^2^, the photoelectrochemical performance of the second alloy film, WFeCu_ox_ displayed photocurrents equal to 0.025 mA/cm^2^ at 1.1 V. Upon increasing the potential of the electrode above 1.3 V under solar illumination, the WFeZn_ox_ film exhibited higher recorded water oxidation photocurrent, which rose to reach 0.3 mA/cm^2^ at 1.8 V vs RHE before the onset of the dark current. It should also be noted that the photocurrent density observed for WFeZn_ox_ here is clearly higher than that for WFeCu_ox_ (0.2 mA/cm^2^ at 1.8 V vs RHE).

To check photoelectrochemical oxidation (under illumination with visible light) at the optimized photoanode (a ternary metal oxide semiconductor composite layer) driving oxygen production, an oxygen membrane sensor—Clark electrode—was introduced in the close vicinity of the photoanode. The measurements were carried out during the photooxidation of water on a WFeZn_ox_ photoanode at an applied potential of 1.2 V under simulated solar AM 1.5 G illumination. The recorded profile is shown in Figure S9, where the value of O_2_ production was detected with increasing durations of photoelectrolysis. The tested WFeZn_ox_ photoanode cleaves water into oxygen with a 30% conversion efficiency.

Figure 10 demonstrates the incident photon-to-current conversion efficiency (IPCE) data characteristic of both WFeZn_ox_ and WFeCu_ox_ films examined in acidic media. The IPCE parameter was found by dividing the obtained photocurrent (measured after subtracting the typically very weak dark current) by the photon flux. It should be noted that for both alloy samples, appreciable photocurrents were recorded at wavelengths below 500 nm. The IPCE approaches a maximum of approximately 10% around 380 nm and then decreases monotonously. A determinable IPCE at this wavelength implies that photogenerated holes can move across nearly the entire length of the layer. Furthermore, it should be noted that the measurable IPCE for WFeCu_ox_ is slightly lower than the IPCE obtained for WFeZn_ox_.

**Figure 10:**
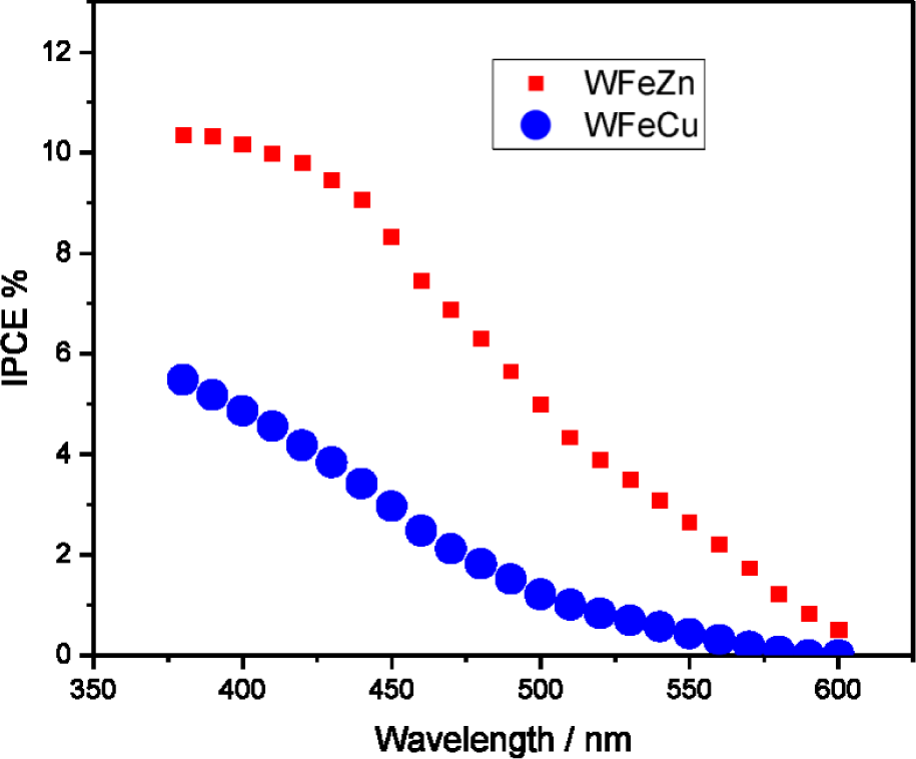
Incident photon-to-current efficiency for WFeZn_ox_ and WFeCu_ox_.

To evaluate the photocatalytic activity of the proposed ternary alloys toward the photocatalytic degradation of organic pollutants, methylene blue (MB) was tested as a model organic pollutant. A photodegradation pathway of MB under visible light is well-known in the literature and leads to products that are still harmful but are no longer absorbed in the visible range of light.^44^ The UV–vis spectra before irradiation show two characteristic peaks in the visible range (the red light range attributed to the presence of MB). However, the UV–vis spectra of the examined solution altered significantly after 90 min of sunlight illumination. In the presence of both ternary alloys, the recorded peaks decreased significantly after 90 min, indicating the degradation of MB. A quantitative analysis of these photocatalysts is presented in Figure 11 which depicts the relation of concentrations (C/C_0_) of MB as a function of illumination time. More precisely, Figure 11a shows the degradation efficiency with no potential applied, whereas Figure 11b shows the same at a +1 V potential. The outcomes demonstrate that the model compound was degraded in the presence of both ternary alloys via photosensitized degradation. The kinetic rate constants and the removal efficiency were found to vary among the different photocatalytic materials. When the electrode was modified with WFeZn_ox_, the pseudofirst order rate constant (*k*) was 0.05 min^–1^ with a total removal of 75% after 90 min of illumination. Degradation of MB at the WFeCu_ox_ electrode was much slower (*k* = 0.03 min^–1^) with a total removal of 50%. The degradation was more efficient with a +1 V potential applied to the electrode.

**Figure 11:**
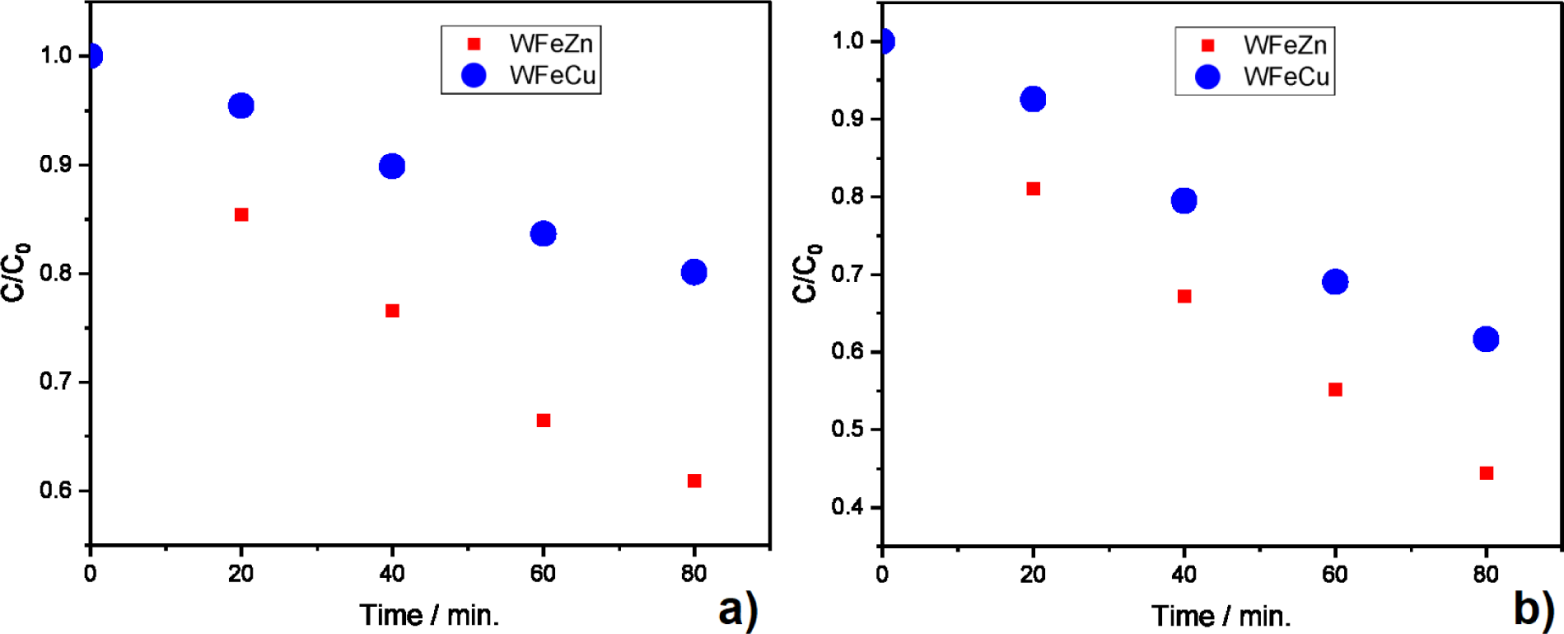
MB photodegradation efficiency of WFeZn_ox_ and WFeCu_ox_ at (a) 0 V and (b) +1 V.

To determine information about the role of electrons and holes in the photodegradation efficacy of methylene blue (MB) by WFeZn_ox_, measurements were performed by evaluating the degradation activities of methylene blue in the presence of selected scavengers. It is commonly accepted that three main active species—holes (h^+^), superoxide radicals (•O2^–^), and hydroxyl radicals (•OH)—are primarily responsible for the photodegradation of various organic pollutants in water samples.^45^ Here, 1 mmol EDTA was utilized as a hole (h^+^) scavenger, and the availability of electrons was expected to increase, allowing the formation of •O2^–^. It was noted that the introduction of EDTA (h^+^ scavenger) led to an 85% decrease in the performance of MB photodegradation. These results indicated that holes (h^+^) are not crucial reactive species in this process. The addition of KBrO_3_ (at a concentration of 1 mmol) as an electron scavenger also caused the reduction of O2 into •O2^–^ radicals, which participate in degrading MB. When KBrO_3_ was used as an electron (e^–^) trap, the photodegradation of MB decreased by around 15%, indicating that electrons (e^–^) play a significant role in the degradation of MB. On the contrary, the introduction of isopropyl alcohol as a hole scavenger exhibited less photocatalytic degradation performance in comparison to electron scavengers. Based on these outcomes, it can be determined that •OH radicals and electrons (e^–^) are the crucial reactive species during the photodegradation of MB.

### Discussion Summary

Optimal conditions for producing WFeZn_ox_ and WFeCu_ox_ layers on FTO, in terms of photoelectrocatalytical activity, are summarized in Table 1.

**Table 1:** Optimal Conditions for the Production of WFeZn_ox_ and WFeCu_ox_ Catalytic Layers

Precursor alloy	WFeZn	WFeCu
Annealing temperature	600 °C	
Annealing duration	60 min	
Fe(III) concentration	27 mmol·dm^–3^	
Zn(II) concentration	2 mmol·dm^–3^	–
Cu(II) concentration	–	1 mmol·dm^–3^
Butynediol concentration	50 ppm	150 ppm
Deposition current density	35 mA·cm^–2^	18 mA·cm^–2^
Deposition temperature	45 °C	25 °C
Deposition duration	3 min	5 min
Estimated layer thickness	240 nm	130 nm

The alloys are electrodeposited under these conditions with a Faradaic efficiency of 16% for WFeZn and 14% for WFeCu, which is comparable to the electrodeposition FE of similar tungsten alloys.^46^

Composition of the materials deposited under optimal conditions is presented in Table 2. The composition of the alloys has been thoroughly examined using EDS prior to further experiments. The composition of the oxidized materials was calculated based on a well-justified assumption that the metals forming the layer fully oxidize to their highest oxidation states. As concluded later, these alloys are likely to convert into tungstate forms through annealing, which does not affect the elemental composition.

**Table 2:** Elemental Composition of the Materials

Material	at. % W	at. % Fe	at. % Zn	at. % Cu	at. % O
WFeZn	18.1%	75.0%	6.9%	–	–
WFeZn_ox_	6.6%	27.4%	2.5%	–	63.5%
WFeCu	23.4%	71.0%	–	5.6%	–
WFeCu_ox_	8.3%	25.1%	–	2.0%	64.6%

The calculated band gap of the material, 2.1 eV, is comparable to the band gap of hematite, which is most likely to be a main component of both layers. Apart from the XRD patterns (Figures 7 and S5), which are the main evidence, the very composition of the alloys also proves this indirectly. For iron constituting 70–80 at. % of the material, even after an efficient conversion of tungsten­(VI) along with iron­(III), zinc­(II), or copper­(II) to compound tungstates, the iron oxide should remain the main component of the layer. The formation of tungstates can be perceived as strongly advantageous for the photocatalytic performance of the final material, especially since the utilization of the tungsten alloy deposition technique for the first step of the synthesis allowed the necessary annealing temperature to be decreased low enough not to destroy the FTO glass substrates.

The metallic precursors for the studied layers, utilized for photoelectrocatalysis, have been proven to be deposited more effectively at relatively low temperatures and, for WFeCu, also at relatively low current density compared to those commonly used for tungsten alloy plating. The divergence from usual values is explained by the difference in applications. Tungsten alloy layers are usually considered protective coatings, which need to be smooth, whereas for catalysts, a rougher surface is more appropriate. A decrease in temperature not only makes the surface of the deposit more developed but also lowers the cost of synthesis. Moreover, the alloy’s corrosion resistance or hardness is irrelevant if the alloy is utilized as a precursor for obtaining oxidized layers. Also, no direct correlation between tungsten content and photoelectrocatalytic efficiency was observed, whereas for obtaining protective coatings, the main trend is to maximize the tungsten content in the alloy.

Out of the two catalysts described herein, WFeZn_ox_ acted more efficiently than WFeCu_ox_ in most conducted experiments, including light-driven oxygen evolution, organic pollutant degradation, and incident photon-to-current efficiency.

## Conclusion

Semiconducting materials containing ternary metal oxides, prepared by electroplating ternary alloy layers on FTO conductive glass from appropriate baths followed by annealing in an oxygen atmosphere, demonstrated photocatalytic properties under solar light irradiation in acidic media. Moreover, the obtained n-type semiconducting electrodes can decompose organic pollutants as well as generate an anodic photocurrent related to the oxidation of water to oxygen. The catalytic efficiency for methylene blue degradation was assessed under visible light illumination. The structure of the alloy obtained by codeposition favors the formation of tungstate phases, which are more efficient catalysts for the aforementioned processes. The presented photoanodes are capable of splitting water into oxygen with 30% efficiency. These properties justify our current approach to producing photoelectrocatalysts for the decomposition of various organic pollutants and the photooxidation of water, based on heat treatment of electroplating-synthesized ternary alloy nanocomposite films.

## Supplementary Material



## Notes

Table containing all the details of the flagged XRD patterns; additional figures, including LSV curves from the optimization process, detailed XRD patterns, and Tauc plots for the materials (PDF)

## Abbreviations

WFeZn: tungsten–iron–zinc alloy
WFeZn_ox_: annealed WFeZn
WFeCu: tungsten–iron–copper alloy
WFeCu_ox_: annealed WFeZn
FE: Faradaic efficiency
LSV: linear sweep voltammetry
OER: oxygen evolution reaction
XRD: X-ray diffractometry
FTO: fluorine-doped tin oxide
MB: methylene blue
RHE: reversible hydrogen electrode
