# Supplementary material for: Photocatalytic Activity of Thin Layers Obtained via Electrodeposition and Annealing of Nanostructured WFeZn and WFeCu Alloys
Source: ACS Omega. 2025 May 15;10(20):20236–46. doi: 10.1021/acsomega.4c11131 (PMC12120569; doi:10.1021/acsomega.4c11131)
Supplement: Supplementary file 1 [file ao4c11131_si_001.pdf]

Supplementary information

Photocatalytic activity of thin layers obtained via electrodeposition and annealing of nanostructured WFeZn and WFeCu alloys

Tomasz Ratajczyk*, Krzysztof Miecznikowski, Pawel Majewski, Rafal Maciag, Mikolaj Donten

University of Warsaw, Faculty of Chemistry. Pasteura 1, 02-093 Warsaw, Poland

The additional data presented in the supplementary information include several types of plots and a table. Figures S1 to S4 cover data collected during the optimization procedures, mostly the LSV curves. Additionally, to the curves recorded for layers deposited at different metal ion concentrations (Fig. S1), relations between the ion concentrations and tungsten content are depicted in Fig. S2. Further, Fig. S3 collects LSV curves depending on temperature of electrodeposition, and S4 collects curves depending on the current density of the deposition process. These plots are intended to expand the view of the experiments on layer optimization. Figures S5, S6 and S7 are an extension to the PXRD data, represented in the main article by pattern for $WFeZn_{ox}$. Fig. S5 shows analogous graph for the other material, $WFeCu_{ox}$, whereas Fig. S6 and S7 present the patterns for both alloys with the predicted reflexes marked for particular phases detected in the studied materials. The summary of crystallographic parameters of compounds used in phase identification for the materials. Fig. S8 contains Tauc plots, mentioned in the main article,

a graphical representation of the band gaps for both $WFeZn_{ox}$ and $WFeCu_{ox}$. Additionally, Fig. S9 depicts profile of photooxidation in the described conditions, measured with utilizing a Clark electrode.

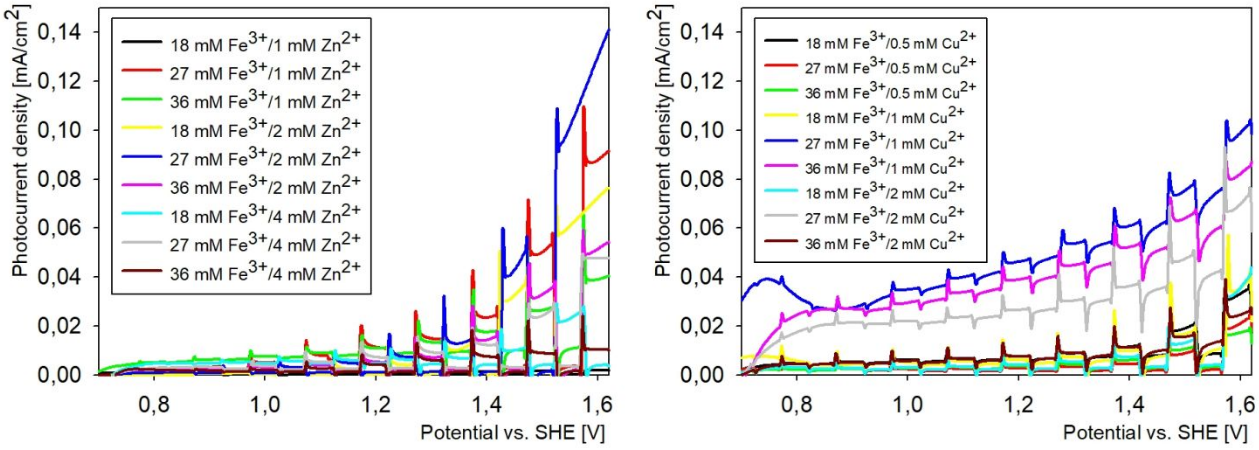

Figure S1. LSV curves for oxidized layers of WFeZn (left) and WFeCu (right) deposited at various concentrations of iron(III), zinc(II) and copper(II) in the bath.

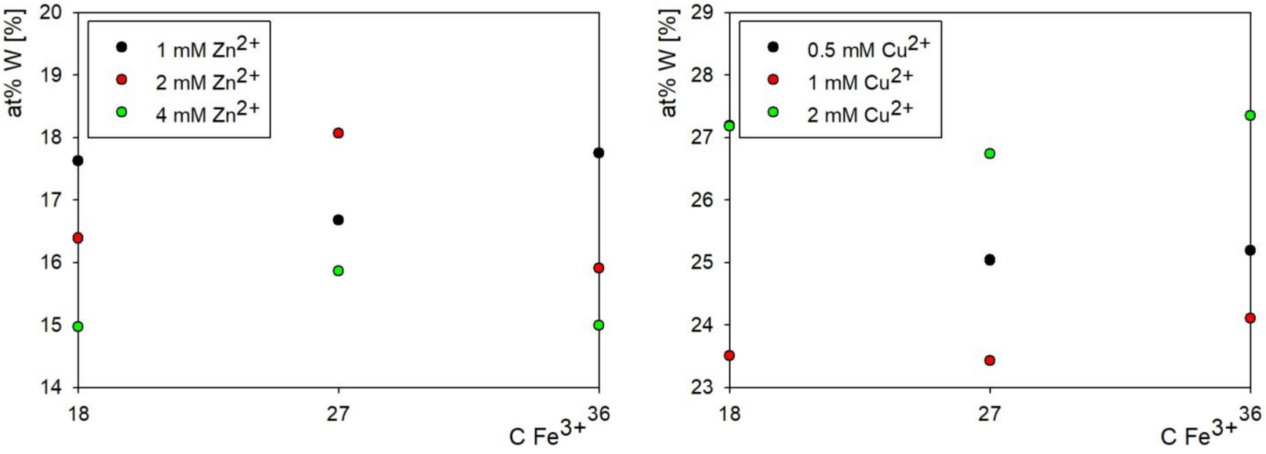

Figure S2. Tungsten content in the alloys corresponding to Fig. S1.

Data presented in Fig. S1-S2. show some some vague, but noticeable correlation between tungsten content in the material and the photocurrent on the final oxidized layer. High-tungsten samples of $WFeZn_{ox}$ tended to exhibit the best photocatalytic performance, whereas for the other material, $WFeCu_{ox}$, better photocurrents were observed for the samples containing less tungsten. In any case, in the optimization procedure, the best performing catalysts have been chosen for the next steps, regardless of trends in their composition.

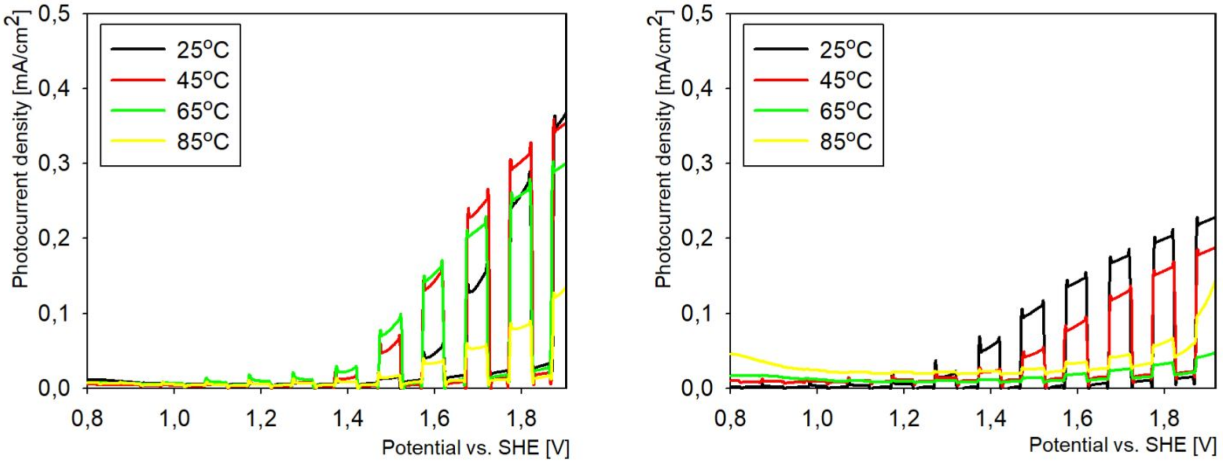

Figure S3. LSV curves for WFeZn (left) and WFeCu (right) deposited at various temperatures

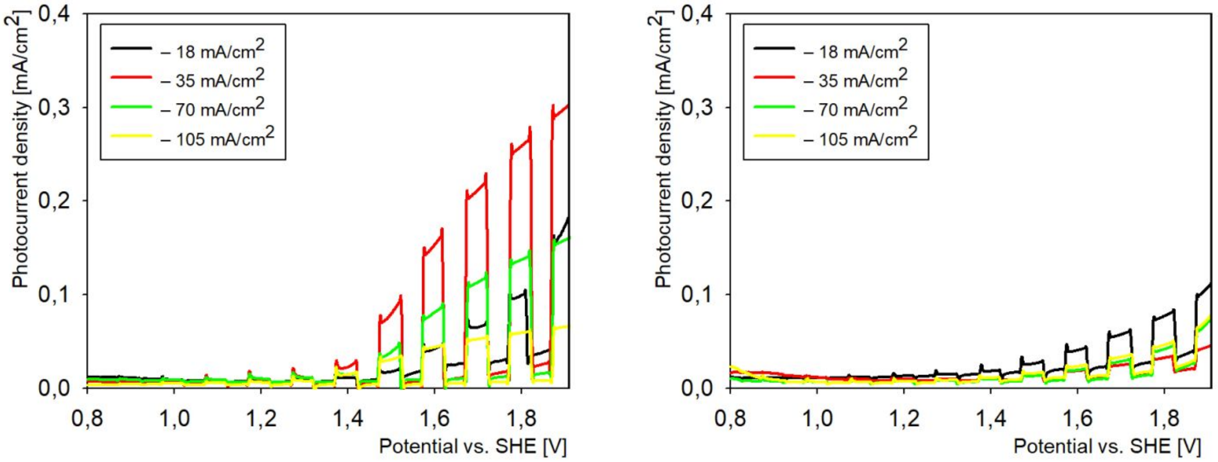

Figure S4. LSV curves for WFeZn (left) and WFeCu (right) deposited at various current densities.

In general, better performing WFeZn and WFeCu layers were obtained from alloys deposited in relatively low temperatures, with relatively low current densities, when compared to conditions commonly utilized for induced codeposition of tungsten [46]. Apart from the still unclear influence of those parameters on any nanostructural details of the outcoming materials, a strong correlation is known, that such low temperatures and current densities would yield alloys of relatively low tungsten content. As most of the works on tungsten alloy deposition focus on the metallic material and increasing its tungsten content for sake of hardness, wear resistance or anti-corrosion properties, it is very common in this field to call lower tungsten content far from optimal. Apparently, in the specific case of obtaining an OER catalyst via oxidation of the alloy layer, this trend is reversed.

Table S1. Summary of crystallographic parameters of materials used in phase identification in oxidized alloys

Material	Space group	a (Å)	b (Å)	c (Å)	α (˚)	β (˚)	γ (˚)
α-Fe_2O_3	R-3c (167)	5.038	5.038	13.772	90	90	120
FTO (SnO_2)	P42/mnm (136)	4.737	4.737	3.186	90	90	90
Fe(Zn)WO_4	P2/c (13)	4.820	5.770	5.120	90	90.86	90
$CuWO_4$	P2/c (13)	4.710	5.580	5.170	90	93.62	90
Fe_2WO_6	Pbcn	4.620	5.070	16.970	90	90	90

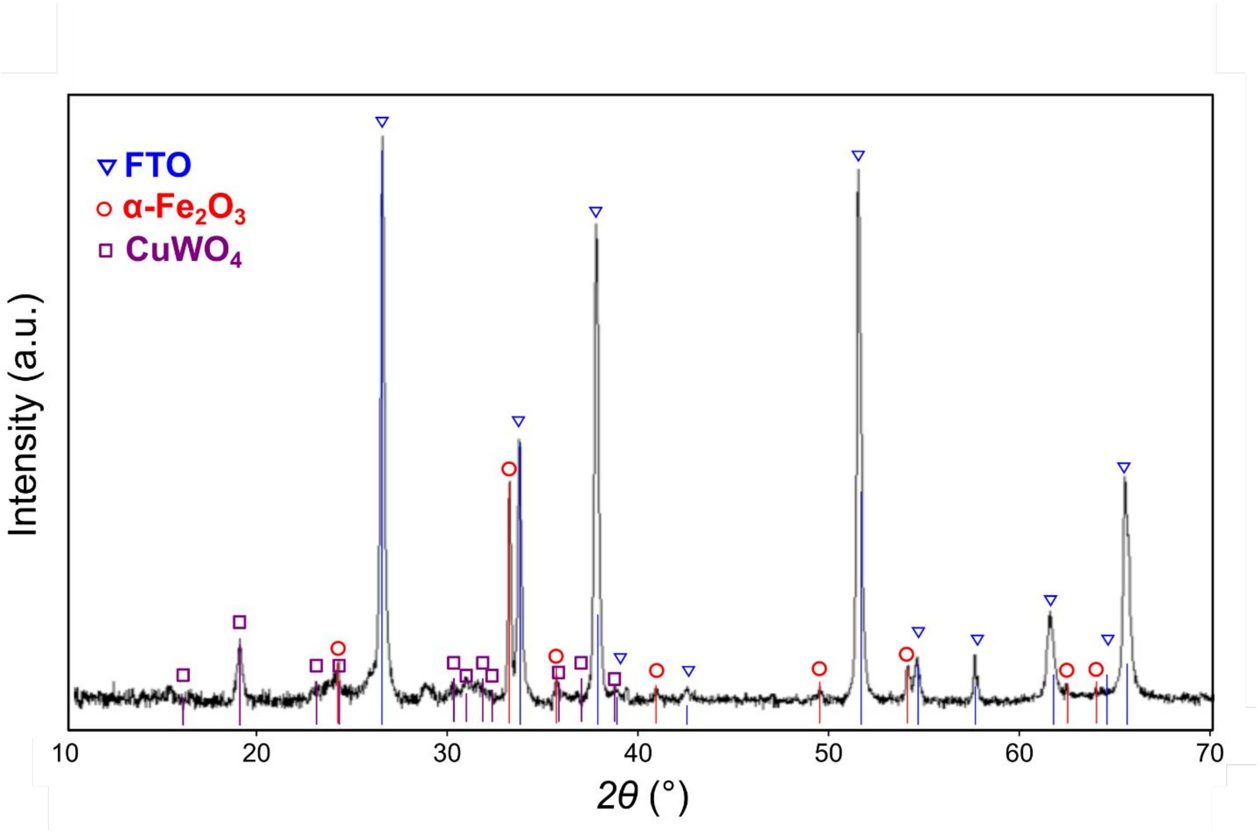

Figure S5. X-ray diffractogram of 130 nm thick $WFeCu_{ox}$Vertical dashed lines and open symbols marked reference positions of the crystalline phases identified in the film. (red circle – α-Fe_2O_3, violet square – $CuWO_4$, blue triangle – FTO)

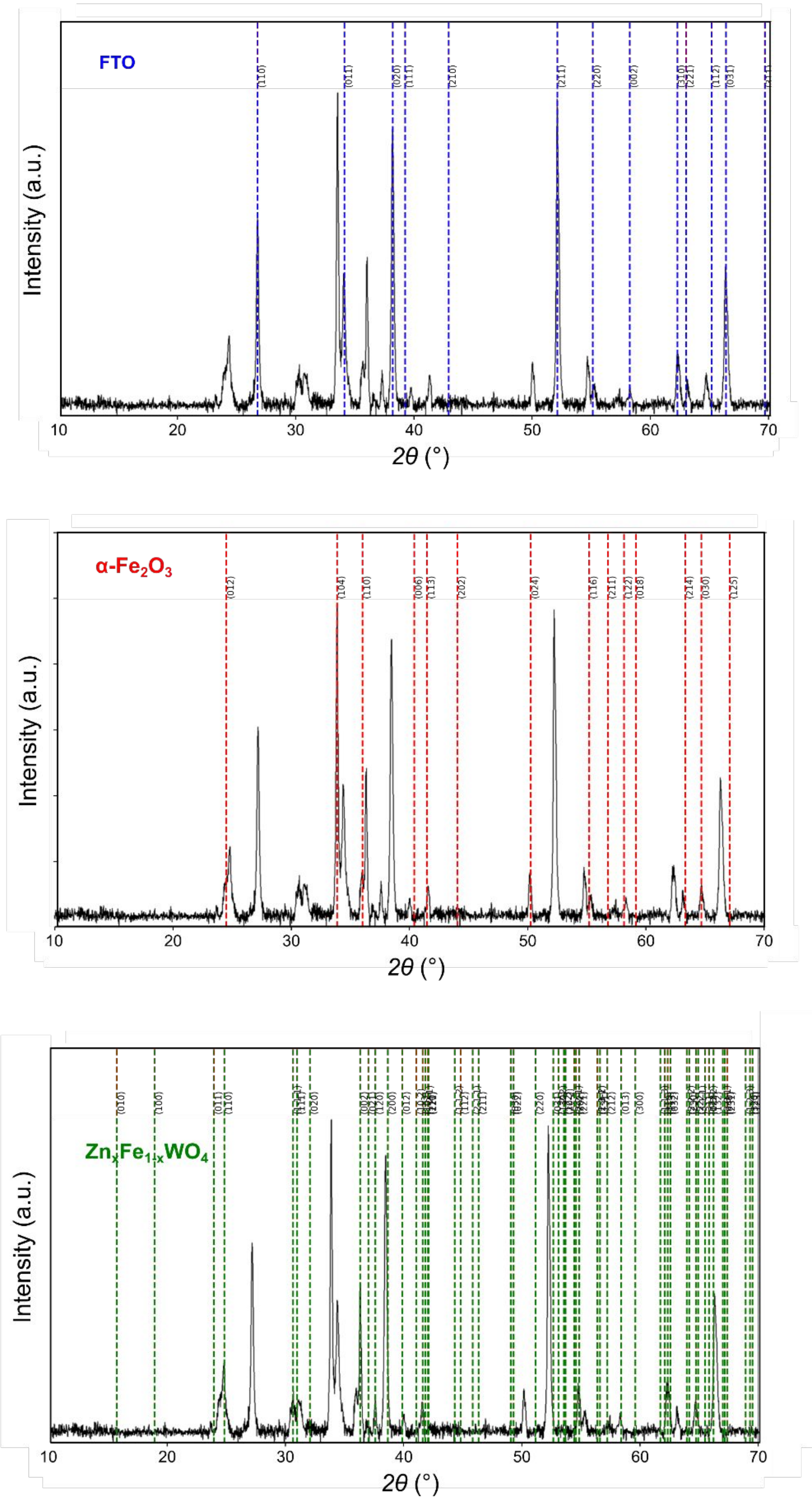

Figure S6. Crystalline phases identified in $WFeZn_{ox}$ (240 nm thick) deposited on FTO

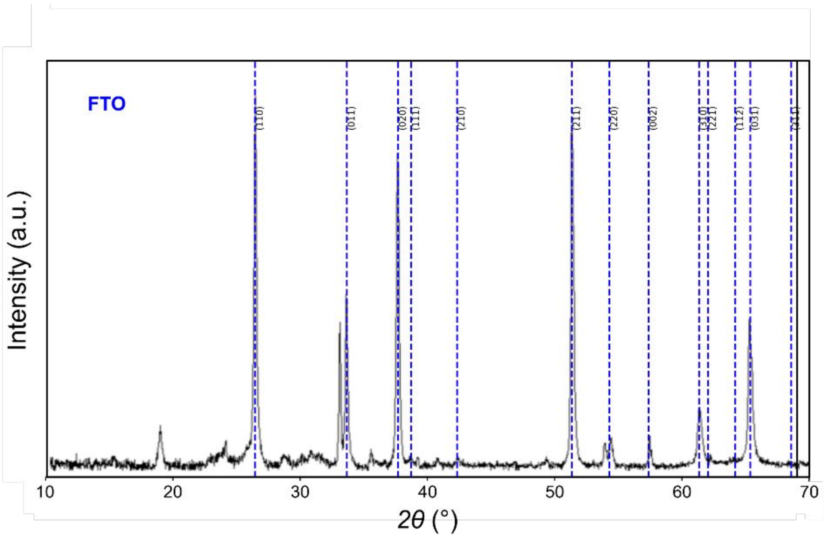
FTO
Intensity (a.u.)
2θ (°)
10 20 30 40 50 60 70

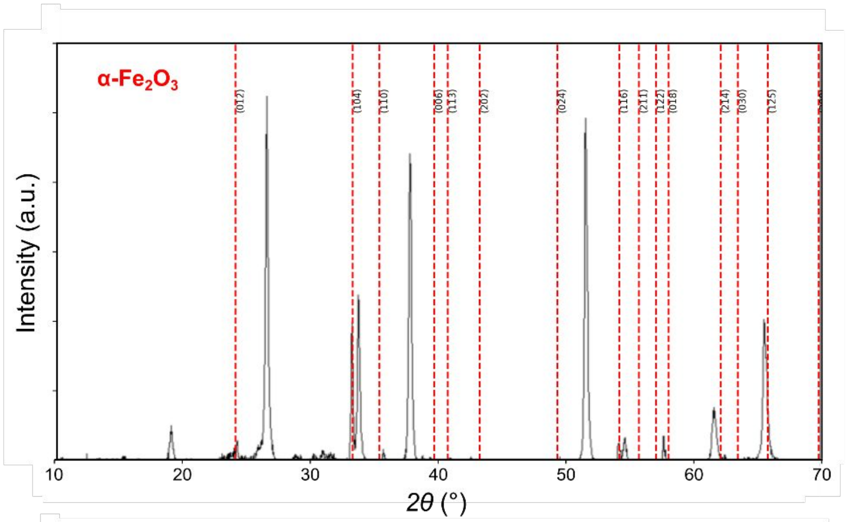
α-Fe_2O_3
Intensity (a.u.)
2θ (°)
10 20 30 40 50 60 70

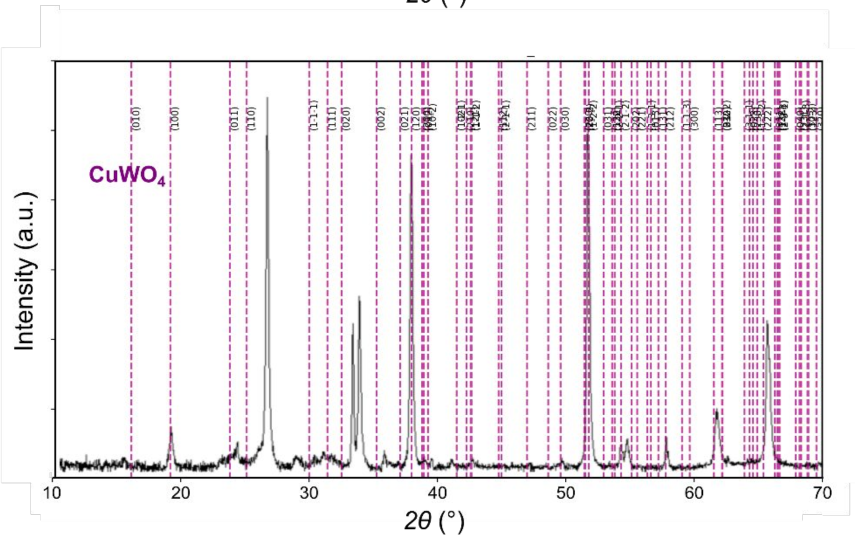
$CuWO_4$
Intensity (a.u.)
2θ (°)
10 20 30 40 50 60 70

Figure S7. Crystalline phases identified in $WFeCu_{ox}$ (130 nm thick) deposited on FTO

The diffractogram for $WFeCu_{ox}$ presented in Fig. S5 is similar to that for $WFeZn_{ox}$, with the main difference being that while iron(II) and zinc tungstates were be treated as isomorphous, copper(II) tungstate has slightly different set of crystallographic parameters and can be marked separately. In both cases, the the diffractograms prove that most of the oxidized tungsten(VI), if not all of it, is converted to suitable tungstate forms during the annealing at 600˚C. The Figure S6-7 exhibit the computed PXRD patterns separately for the plausible components of both materials.

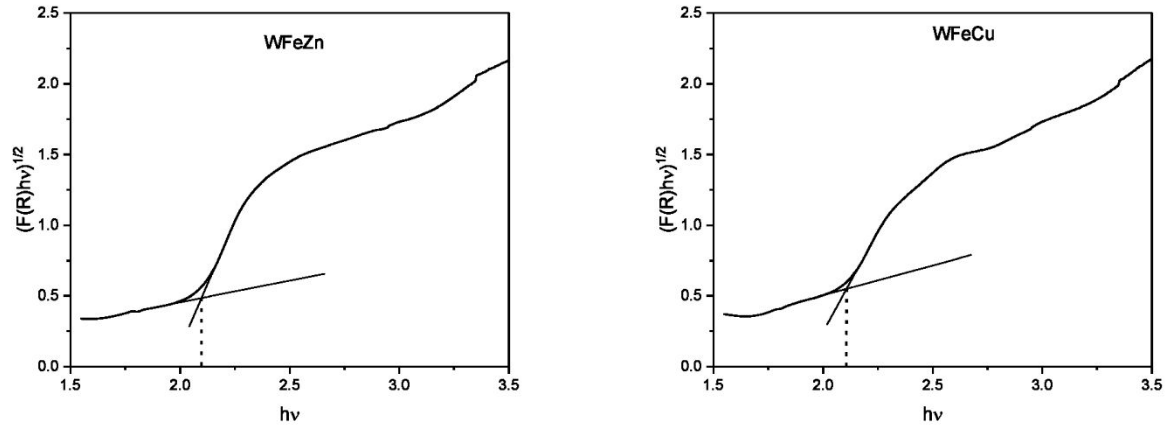

Figure S8. Tauc plots for $WFeZn_{ox}$ (left) and $WFeCu_{ox}$ (right).

The Tauc diagrams, plotted for both the materials, allowed to calculate the band gaps (Fig. S8). For both materials, the band gap is 2.1 eV and can be compared to band gap of hematite. The detailed discussion of this result can be found in the result section of the core article.

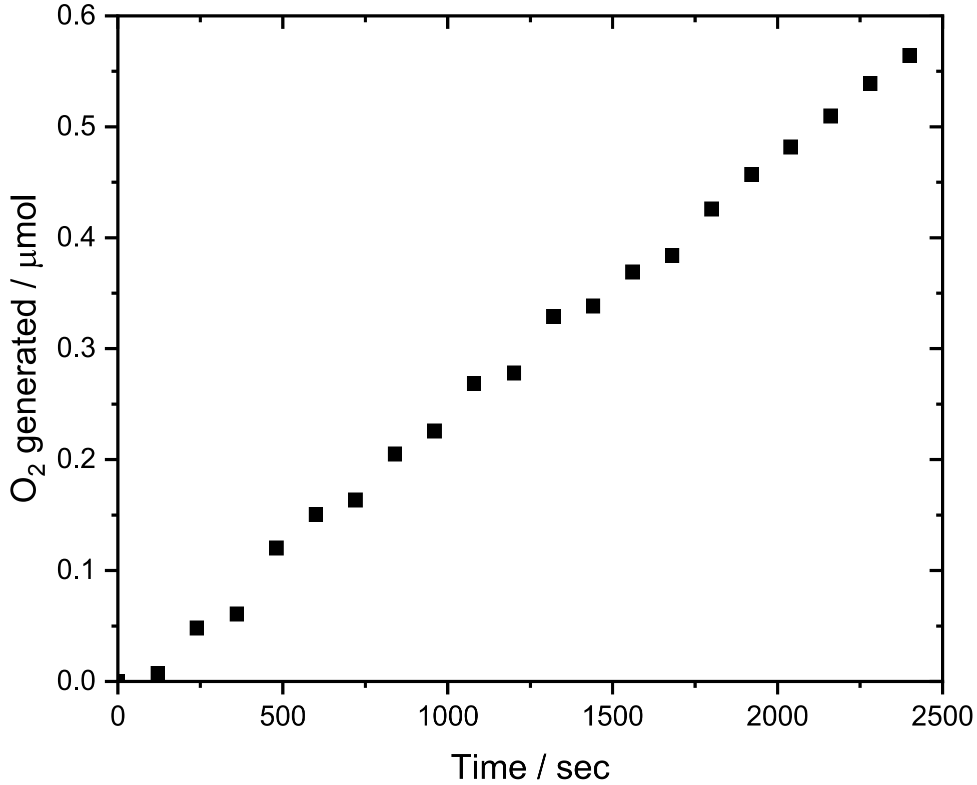

Fig. S9. Monitoring of dissolved oxygen (utilizing Clark electrode) during water splitting on a $WFeZn_{ox}$ photoanode carried out in 0.5 M H_2SO_4 at an applied potential of 1.2 V under the simulated solar AM 1.5 G irradiation.
